# The Acquisition of Orthographic Knowledge: Evidence from the Lexicality Effects on N400

**DOI:** 10.3389/fpsyg.2017.00433

**Published:** 2017-03-30

**Authors:** Yu-Lin Tzeng, Chun-Hsien Hsu, Yu-Chen Huang, Chia-Ying Lee

**Affiliations:** ^1^Institute of Neuroscience, National Yang-Ming UniversityTaipei, Taiwan; ^2^Brain and Language Laboratory, Institute of Linguistics, Academia SinicaTaipei, Taiwan; ^3^Department of Rehabilitation, Chung Shan Medical University HospitalTaichung City, Taiwan; ^4^Institute of Cognitive Neuroscience, National Central UniversityTaoyuan, Taiwan; ^5^Research Center for Mind, Brain and Learning, National Chengchi UniversityTaipei, Taiwan

**Keywords:** lexicality, N400, orthography, reading development, Chinese

## Abstract

This study aimed to understand how reading ability shapes the lexicality effects on N400. Fifty-three typical developing children from the second to the sixth grades were asked to perform the pronounceability judgment task on a set of Chinese real characters (RC), pseudocharacters (PC) and non-characters (NC), as ERPs were recorded. The cluster-based permutation analysis revealed that children with low- to medium-reading ability showed greater negativity to NCs than to RCs and PCs in frontal sites from 300 to 450 ms, while children with high ability group showed a greater positivity to NCs than both RCs and PCs at central to posterior sites. Furthermore, the linear mixed model (LMM) analysis was applied to investigate the relationship between lexicality effects on N400 and reading-related behavioral assessments on a set of standardized tests (including character recognition, vocabulary size, phonological awareness, and working memory). The results found that in children with lower reading ability, the N400 elicited by NCs becomes more negative in the frontal sites. For children with higher reading ability, the N400 elicited by NCs became more positive than that elicited by RCs or PCs in the posterior sites. These findings demonstrate the developmental changes in the lexicality effects on N400 as children become more advanced readers and suggested that the lexicality effects on N400 can serve as neural markers for the evaluation of orthographic proficiency in reading development.

## Introduction

Learning to read is a process of understanding written speech. During early years of literacy acquisition, children’s primary task is to master the orthographic rules that describe a set of orthographic units and mapping principles underlying how orthographic units encode phonology and semantics of a given writing system. For example, English orthography contains 26 letters and the grapheme-to-phoneme corresponding rules for how letters or letter strings (such as “sh,” “th”) are mapped onto phonological units. Studies have suggested that children develop orthographic knowledge gradually throughout the early school years and reach adult levels, at least for simple words, by the fourth grade (e.g., see Stanovich, 1980; Barron, 1981; Adams, 1990).

Studies in alphabetic writing system have used the lexicality effect, which refers to the differences in reading words, pseudowords, and non-words, to examine the development of orthographic knowledge. To be more specific, words and pseudowords are orthographically legal while non-words are orthographically and phonologically illegal random letter strings. Unlike real words, both pseudowords and non-words do not have entries in the mental lexicon. Beginning readers, who have a limited experience with words and thus do not yet master the orthographic rules, may show little difference in processing words, pseudowords, and non-words. As children gain more experience with words and thus develop the orthographic knowledge, they become more sensitive to the concept of “word-likeness” and show less effortful processing for words than for pseudowords and non-words. Indeed, Zoccolotti et al. (2008) reported that the lexicality effect (advantage of reading words over pseudowords) increased with grades and suggested a progressive differentiation in the ability to read words and pseudowords. Other studies also found that first graders process most strings in a letter-by-letter fashion and show no difference between processing of pseudowords and non-words until second or third grade (Rosinski and Wheeler, 1972; Lefton and Spragins, 1974). Most importantly, Allington (1978) found that orthographic sensitivity is a direct function of reading achievement level, but not age or school experience in general. These findings suggest that the concept of “word-likeness” develops throughout the process of learning to read and that the pattern of lexicality effect may change with advancing reading ability.

Previous studies with skilled readers have reported the lexicality effect on N400, an event-related potential (ERP) component to index the semantic retrieval during lexical access (Ziegler et al., 1997; Bentin et al., 1999). For example, Ziegler et al. (1997) measured brain responses elicited by words, pseudowords and non-words during the letter-search or semantic categorization tasks. They found that pseudowords elicited greater N400s than real words, while non-words did not elicit N400s but a greater positivity in later time windows instead. Similar pattern of lexicality effect in adults has also been found in other studies (Nobre and McCarthy, 1994; Bentin et al., 1999). The fact that pseudowords elicited a greater N400 than words has been interpreted as a greater effort elicited by pseudowords during lexical search, as the readers were able to complete the orthographic and phonological processing yet could not find matched semantic representation for pseudowords. The finding also implies that N400 may serve as a “default response” to words or potential words (i.e., pseudowords) (Kutas and Van Petten, 1994). In addition, lack of N400 elicited by non-words suggests that skilled readers can decide that non-words are meaningless strings of letters rather than potential words on the basis of visual/orthographic structure and thus show no attempt to look for semantic representation when encountering non-words.

Coch and colleagues conducted a series of studies by using the lexicality effect on N400 to investigate the development of orthographic knowledge in children (Coch et al., 2002, 2012; Coch and Holcomb, 2003; Coch and Mitra, 2010; Coch, 2015; Coch and Benoit, 2015). For example, Coch et al. (2002) recorded brain responses to real words, pseudowords, non-words, and false fonts in a group of 10- and 11-year-old children. Unlike data from adults showing that words and pseudowords but not non-words elicited centro-posteriorly distributed N400s-, 10–11 year-old children’s data showed anteriorly distributed N400s to all types of stimuli. Although children’s N400 did not show significant effects of word type, planned comparisons revealed greater N400s for words and pseudowords than for non-words and false fonts. Similar findings were also found in younger children with more advanced reading ability. For example, Coch and Holcomb (2003) reported that 7-year-old first graders with high reading ability (scored above the fourth grade level on the standardized reading tests) showed the N400s distributed as described above to known words, unknown words or pseudowords, difficult words, and non-words, whereas low-reading ability group showed no substantial N400 to any type of stimuli. Across these two studies, the marked N400s to non-words and false fonts in normal developing children in 7- to 11-year-old suggest that these children may treat non-words and false fonts as potential lexical items. To further investigate when the concept of word-likeness becomes fully developed, Coch (2015) examined the lexicality effect on N400 in third graders, fourth graders, fifth graders, and adult college students. This study did not replicate the typical lexicality effect in adults. Across the four groups, there was no difference between words and pseudowords, but a greater N400s for pseudowords versus non-words was observed. A difference between non-words and false fonts was found in adults, but not in any children’s groups. Taken together, Coch’s series of studies suggests that the automaticity of orthographic processing to efficiently discriminate the illegal from legal orthographic forms is not fully developed even in the fifth grade. However, it is unclear why college students in Coch’s (2015) study did not show the typical lexicality effect between pseudowords and words and why the three age groups did not show developmental changes in the pattern of lexicality effects on N400.

Previous ERP studies of language acquisition have mainly investigated the developmental changes at a group level. However, children of the same age (or grade) group often show a large amount of individual variability in their reading performance. Averaging the data from a group of children of the same age or grade may obscure important variations at the individual level. Another approach is to examine the relationship between ERP markers and individual reading ability (Henderson et al., 2011; Coch and Benoit, 2015; Khalifian et al., 2015). For example, Coch and Holcomb (2003) reported a significant correlation between N400 amplitudes across all word types and the composite score based on a set of reading ability tests in a group of 14 first graders. Henderson et al. (2011) also reported significant correlations between N400 elicited by pairs of pictures and spoken words and scores on listening comprehension, non-word decoding, and word reading in 8- to 10-year-old children. However, these studies examined the relationship between N400 and reading ability at individual level with a small sample size in a relatively narrow range of age.

Only a few studies have attempted to address the relationship between behavioral assessments and lexicality effects on N400 with a larger sample size. For example, Coch and Benoit (2015) investigated correlational relationships between N400s elicited by real words, pseudowords, non-words, and false fonts and a set of standardized tests for spelling, phonological processing, vocabulary, comprehension, naming, and memory in 72 typically developing, late elementary school children. Among all the behavioral assessments, only vocabulary size (measured by Peabody Picture Vocabulary Test [PPVT]) showed significant correlations with N400s elicited by words (*r* = -0.272) and pseudowords (*r* = -0.235), as higher PPVT scores were associated with more negative N400s. Khalifian et al. (2015) collected ERPs and reading-related behavioral measures from 103 children in grades pre-K to 7. Importantly, they examined the brain-behavioral relationship at individual level by using the linear-mixed effect model (LMM) which allows all random factors (i.e., participants and stimuli factors) to be considered simultaneously with fixed effects of interests, such as behavioral scores and ERPs responses. In addition to replicating the relationship between N400 and vocabulary size, this study also observed a significant relationship between the N250 and academic achievement, as measured by school’s report-card scores. Taken together, the abovementioned studies characterized relationship between ERPs maker and reading outcome at individual level and suggested that ERP measures may have important practical applications in reading intervention.

Chinese, unlike alphabetic writing, is considered as a morphosyllabic writing system (DeFrancis, 1986). The basic written unit of Chinese, the character, is constructed by a combination of stroke patterns or radicals within a constant square-shaped space. The pronunciation of a character cannot be computed sound-by-sound from its constituent parts. Nevertheless, studies have suggested that Chinese readers gradually acquire various kinds of orthographic knowledge, including the statistical properties of radical positions and how radicals convey semantic and phonological information as they learn to read Chinese (Lo et al., 2007; Hsu et al., 2009; Su et al., 2012). However, it remains unclear how reading experience shapes the acquisition of orthographic knowledge in learning to read Chinese. This study aims to address this question by measuring children’s N400 responses to real characters (RC), pseudocharacters (PC), and non-characters (NC) in typical developing children from second to sixth grades.

Based on the database of traditional Chinese characters (Chang Y.N. et al., 2016), over 80% of modern Chinese characters are phonograms, which consist of a semantic component (radical) that provides information about the meaning of a character (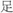, *zú* ‘foot’) and a phonetic component that provides information about the character’s pronunciation (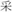, *cǎi* ‘gathering’). The majority of phonogram (around 72%) have a left-right structure with the semantic and phonetic radical standing side by side (i.e., 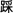 *cǎi* ‘to step on’). Among these phonograms with left-right structure, around 90% have their semantic radicals (S) on the left and phonetic radicals (P) on the right (i.e., 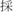, *cǎi*, a SP character), and only 10% have their phonetic radicals on the left side (i.e., 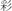, *cǎi*, a PS character). However, the semantic radical and phonetic radicals can also be arranged with the top–bottom structure (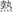, rè ‘hot’), closure structure (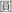, yuán ‘round’), and the semi-closure structure (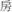, fáng, ‘house’). Given the dominant distribution of SP characters compared with PS characters, studies have shown that Chinese skilled readers develop statistical knowledge about the positions of the radicals in order to retrieve the phonological or semantic information embedded in the characters (Hsiao et al., 2007a,b; Lo et al., 2007; Su et al., 2012).

In addition to radical’s position, a growing body of evidence has shown that Chinese readers utilize a set of orthographic features, including radical combinability (Taft and Zhu, 1997; Hsiao et al., 2007b; Hsu et al., 2009, 2014), the phonetic consistency and regularity (Lee et al., 2005, 2007, 2010; Chang Y.N. et al., 2016), and the semantic transparency of a semantic radical (Chen and Weekes, 2004) for reading Chinese characters. Moreover, children seem to gradually develop different aspects of orthographic knowledge while learning to read. For example, Lo et al. (2007) examined how children name Chinese PCs and found that beginning readers tended to name the pronounceable radical, regardless of its position. As their reading ability increased (measured by the Grade Chinese Character recognition test), high ability children tended to use the radical on the right side to infer PCs’ pronunciations. Tzeng and Lee (2012) tried to track when and how the orthographic knowledge of phonetic radical is developed in children from the fourth to the sixth grades. The data showed that the phonetic consistency effect emerged in children of the fourth grade in reading regular phonograms. The consistency effect was not found in reading irregular phonograms until the fifth grade. By the sixth grade, the consistency effect was found in reading all types of phonograms.

Previous studies have used N170, an ERPs component to index the perceptual expertise of visual word recognition, to investigate the relationship between ERP responses of lexicality effect and Chinese reading development (Cao and Zhang, 2011; Cao et al., 2011; Lin et al., 2011; Zhao et al., 2012). Lin et al. (2011) reported that, relative to false-characters and stroke combinations, real- and PCs evoked greater N170 in the left posterior brain region in adults. As for children, Cao and Zhang (2011) reported a greater N170 for character over stroke combination in the sixth grade children, but not in the second grade and thus suggested that the subtle lexicality effect on N170 had well developed in children by the sixth grade after 5 years of normal school reading training. Very few studies have addressed this issue by using N400. Chung et al. (2012) examined whether third to fourth grade children with Chinese developmental dyslexia and their age-matched controls showed different patterns of the lexicality effect on N400. Participants were asked to read a set of RCs, PCs (novel combination of a phonetic radical and a semantic radical), and NCs (a mirror image of a RC). Congruent with findings of alphabetic writing systems, both groups showed marked N400s to all three types of stimuli. For typically developing children, PCs elicited a greater N400 than RCs and there was a graded lexicality effect on N400 (pseudocharacters > real characters > non-character). However, dyslexic children showed no difference among the three types of stimuli. They thus suggested that Chinese dyslexia may be characterized by a deficit in processing orthographic information.

The present study aimed to investigate how reading ability shapes two types of lexicality effects on N400 in learning to read Chinese. To achieve the goal, we conducted the cluster-based random permutation analyses (Maris and Oostenveld, 2007) to characterize the temporal dynamics and topographic distributions of lexicality effects in children with different levels of reading ability. Thus, the 53 typically developing children were subdivided into three groups based on their reading ability. To further investigate the relationship between lexicality effects on N400 and reading-related behavioral assessments on a set of standardized tests (including character recognition, vocabulary size, phonological awareness, and working memory), the LMM analysis was applied with ERP data from every child instead of dividing children into groups. It was expected that as children gained more experiences with Chinese character reading, they would become more efficient in discriminating legal (characters and pseudocharacters) and illegal (NCs) orthographic forms. In other words, children with higher reading ability were hypothesized to show less negative N400 for NCs. Thus, an interaction was expected to be found between reading ability and the lexicality effects, by contrasting the N400 of NCs to that of RCs and PCs, respectively.

## Materials and Methods

### Participants

Fifty-three elementary school children (26 girls) aged from the second grade to the sixth grade (number of children in each Grade: Grade2 = 4, Grade3 = 14, Grade4 = 7, Grade5 = 11, Grade6 = 17) participated in this experiment, with average age of 10.73 years (range7.44∼12.78, *SD*: 1.5). All children were with normal non-verbal intelligence and were assessed by five subtests of the Wechsler Intelligence Scale for Children-Third Edition (WISC-III) in Chinese version (Chen and Chen, 1997), including Coding, Arithmetic, Block design, Digit span and Maze. These children were divided into three reading-ability groups by their GCCRT scores (sample size in each group, high: 19, medium: 18, low: 16) for the cluster-based random permutation analyses. The characteristics and behavioral assessments of three reading-ability groups were summarized in **Table 1**. All participants had normal or corrected to normal vision and no history of psychiatric or neurological disorders. This study was approved by the Institutional Review Board of Academia Sinica. Informed consent forms were obtained from all children and their parents.

**Table 1:** The results of behavioral tests.

	High ability	Medium ability	Low ability
N	19	18	16
Age	11.6 (1.2)	10.8 (1.3)	9.6 (1.2)
GCCRT	9.2 (0.48)	6.9 (0.70)	4.5 (0.98)
PPVT	119.8 (15.89)	115.3 (14.15)	117.4 (14.33)
PA	14.1 (1.62)	12.6 (3.11)	12.4 (4.01)
RAN_Digit	15.14 (3.93)	17.22 (4.21)	18.26 (3.80)
RAN_ZYFH	27.45 (6.60)	29.55 (8.12)	32.55 (9.44)

*GCCRT, the Grade Chinese Character Recognition test. PPVT, the Chinese version of Peabody Picture Vocabulary Test, Revised. PA, Phonological awareness test. RAN_Digit, Rapid automatized naming in digits (in second). RAN_ZYFH, Rapid automatized naming in Zhuyin FuHao (in second). Standard deviations are given in parentheses.*

### Stimuli

#### Standardized Behavioral Assessments

Participants were given a battery of standardized tests to measure their general cognitive ability and reading-related skills. Five subtests of the Wechsler Intelligence Scale for Children-Third Edition (WISC-III) in Chinese version (Chen and Chen, 1997), including Coding, Arithmetic, Block design, Digit span and Maze, were used for the assessment of non-verbal intelligence. The Chinese version of the Peabody Picture Vocabulary Test, Revised, PPVT-R (Lu and Liu, 1998) was used to provide a measure of receptive vocabulary. The Graded Chinese Character Recognition Test (GCCRT) (Huang, 2001) consists of 200 Chinese characters which can be subdivided into 10 levels according to the frequency of use. This test is frequently used in Taiwan to estimate children’s reading ability in grade 1–9 and for the diagnosis for Chinese dyslexia. The test requires the participants to name the characters from easy to difficult and was stopped when a participant would incorrectly name ten consecutive characters. The total number of correctly answered characters can be transformed into a standardized score for the grade level of Chinese character precognition ability. In addition to GCCRT, every participant was examined using Chinese Phonological Awareness Test (PA) (Tzeng et al., 2006) and Rapid Automatized Naming test (RAN) (Tzeng et al., 2011) for assessing their phonological sensitivity and decoding ability. Children were allowed to take breaks between the tests as long as they needed. The administration of all the tests took 70–90 min.

#### Experimental Stimuli

**FIGURE 1:**
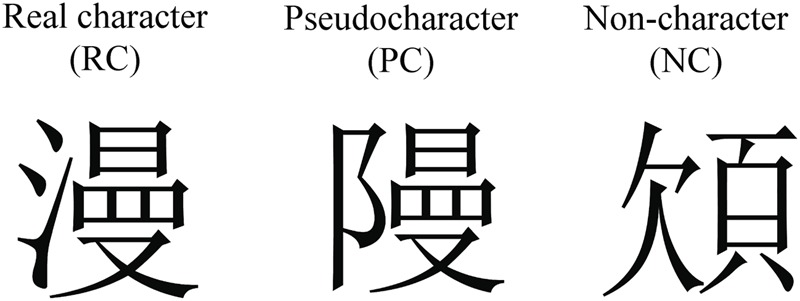
**Example stimuli for real characters, pseudocharacters, and non-characters used in this study**.

Stimuli for the ERP experiment consist of three types: RC, PC, and NC (see examples in **Figure 1**). The first type of stimuli, the real character, consisted of 60 traditional Chinese phonograms that were selected from the Chinese textbooks that used in Taiwanese elementary schools. Two-thirds of the real characters contain a semantic radical on the left side and a phonetic radical on the right side. The remaining one-third of real characters contain the reversed internal structure, a semantic radical on the right side and a phonetic radical on the left side. According to the Survey of the characters and words frequently used by primary school students (Ministry of Education, 2002), the mean frequency of real characters is 30.90 (±24.07) per million words. The second type of stimuli are PCs were made up from the same set of real characters by replacing their semantic radicals while maintaining the same phonetic radicals in their position. Therefore, PC was constructed following the orthographic rule while representing a novel combination of a semantic radical and a phonetic radical and thus they are orthographically legal and phonologically pronounceable, yet meaningless orthographic forms. The third type of stimuli were NCs. Each NC consisted of two constitutes standing side by side to remain the same left-right structure. One of the constitutes is an illegal radical that was created by deleting or adding one stroke to the legal phonetic or semantic radical. The other one is a legal radical but placed in the illegal radical position. The radicals that were placed in the illegal position were chosen from the database of traditional Chinese characters (Chang Y.N. et al., 2016) and half of them were semantic radicals that could only appear on the left and the other half were phonetic radicals that could only appear on the right of Chinese phonograms. Thus, all NCs are unpronounceable, meaningless and illegal orthographic structure. These three types of stimuli were matched for the number of strokes [*F*(2,167) = 0.72, *p >* 0.05].

### Procedure

Before signing the consent form, the participants and their parents were first given a tour of the laboratory and an overview of the experiment. Every participant would first undergo a set of behavioral tests and then the ERP experiment.

For the ERP experiment, participants were seated in front of a monitor, at a distance of approximately 60 cm, to perform a pronounceability judgment task. Each participant was given 24 trials for practice and 180 randomized experimental trials in five sessions. Participants were allowed to take a break between sessions for as long as they required. A trial began with the presentation of two vertical lines in the center of the screen for 500 ms. Then, the target was presented between the two lines. These stimuli would remain on the screen until the participants make a response. Participants were informed that they might encounter many unknown characters or character-like stimuli and their job was to decide or guess whether the presented stimuli were pronounceable or not by pressing the right or the left button on a response box for “pronounceable” and “unpronounceable,” respectively, as quickly as possible. After processing a button, the stimulus was replaced with a fixation cross in the center of the screen for 500 ms. A capital letter ‘B’ was then displayed for 1000 ms to instruct the participants that they may blink their eyes during this period of time, if necessary. The pronounceability judgment task was designed to ensure that children would engage reading process to the stimuli while they would not be discouraged by the a large number of unknown stimuli during the experiment, especially for young children with relatively low reading ability.

#### EEG Recording and Data Analysis

Continuous electroencephalogram (EEG) was recorded from 32 Ag/AgCl electrodes (QuickCap, Neuromedical Supplies, Sterling, VA, USA). The electrodes were online referenced to the average of left and right mastoids. Vertical and horizontal eye movements were recorded by electrodes placed on the supra- and infraorbital ridges of left eye (VEOG), and the outer canthi of the left and right eyes (HEOG). Electrode impedance was kept below 5 KΩ. The EEG signal was amplified by SynAmps2 (Neuroscan, Inc.) in DC mode, low-pass 100 Hz and digitized at a sampling rate of 1000 Hz.

All trials were used in ERPs preprocessing and further statistical analysis, except for trials with response time (RT) shorter than 200 ms. For off-line analysis, the EEG signal was segmented into epochs with 100 ms pre-stimulus onset and lasting 900 ms post-stimulus onset. Children’s ERPs used to have low signal-to-noise ratio (SNR). Previous studies have suggested that the ensemble empirical mode decomposition (EEMD) method can improve remarkably the SNR during measurements of ERPs (Cong et al., 2010; Al-Subari et al., 2015a,b; Chen et al., 2016; Hsu et al., 2016). For example, Hsu et al. (2016) applied EEMD to reanalyze the dataset of Cheng et al. (2013) and demonstrated that only one third of the original trials were required to replicate the mismatch negativity (MMN) effect with the approximate effect size. Chen et al. (2016) also used the same analytic procedure for N400 measurement. Thus, the present study also applied the analytic procedure that has been described in Hsu et al. (2016) for data analysis. The time range of EEG segments for EEMD analysis was from 200 ms before the stimuli onset to 1000 ms after the onset. The EEMD analysis was performed with 10 times of sifting and 40 ensembles. The amplitude of Gaussian noises used in the EEMD procedure was 10 percentage of EEG signal’s standard deviation. Each EEG segment was decomposed into eight Intrinsic mode functions (IMFs). IMF8 was the residual trend and only IMFs 1–7 could be considered to estimate the event-related Modes (ERMs). Subsequent analyses were based on IMFs between -100 ms and 900 ms. According to previous studies, the summation of IMF6 and IMF7 was used to extract the N400 signal. Hilbert spectra of these IMFs revealed frequencies ranging from 2 to 6 Hz and from 1 to 4 Hz, respectively. Then, waveforms of ERMs were calculated by averaging IMFs over trials and were performed for each stimuli condition and each channel and each participant.

### Statistical Analysis for Behavioral Data and N400

#### Behavioral Data

Response times and response preference were analyzed by using LMM models described below. Participants were divided into three groups based on their GCCRT scores to examine how behavioral responses change with reading ability. In the model, the assigned group (high, medium, and low) and lexicality (RC, PC, and NC) were specified as the fixed effects. Sliding contrasts (high-medium and medium-low) were used to evaluate the group effects. To fulfill the requirement of linear independency of the designed contrasts (Kutner, 2005), the contrast of lexicality was specified as +0.5 and -0.5 for both the NC-PC and NC-RC effects. The two-way interaction between each contrast and group effect was also examined. For RT, statistical inferences about effects of lexicality and group are based on LMM. As for response preferences, the data was analyzed with generalized LMM (Bolker et al., 2009). Responses for pronounceable and unpronounceable were scored as 1 and 0, respectively.

#### ERPs Data: Cluster-Based Permutation Analysis

**FIGURE 2:**
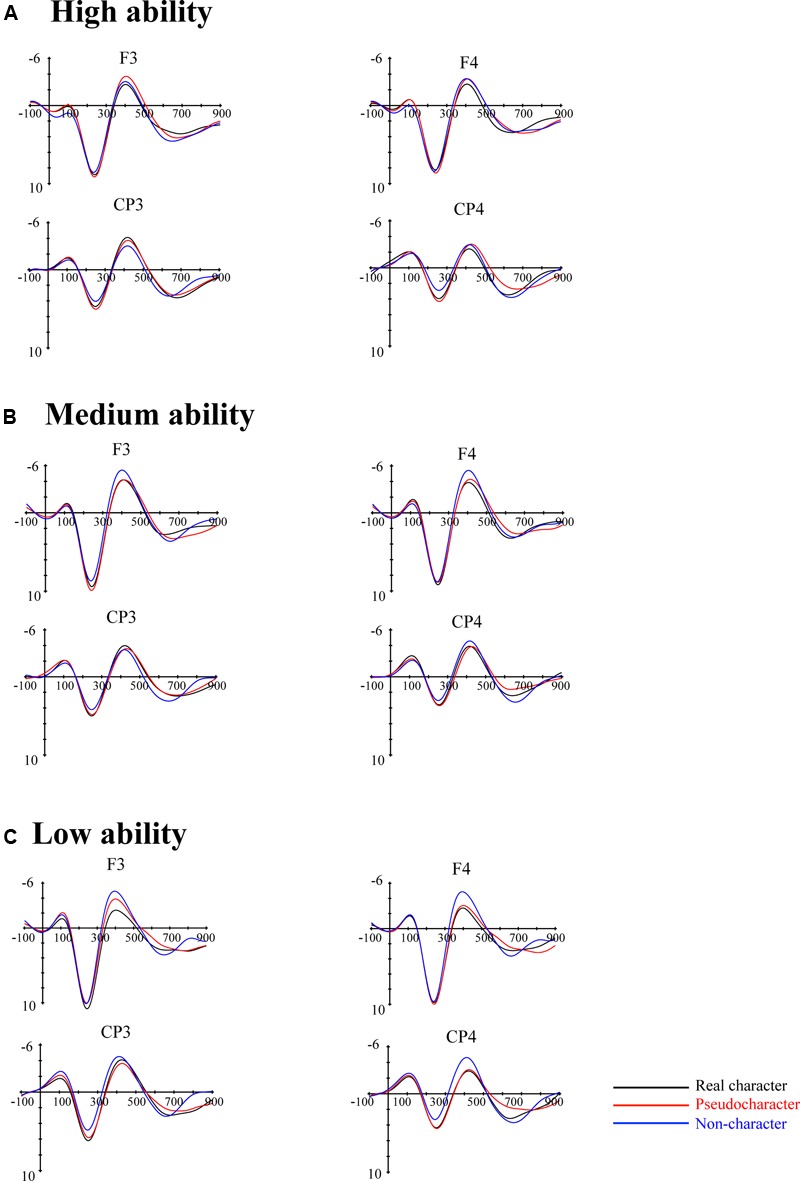
**Grand averaged ERMs for real character (black line), pseudocharacter (red line) and non-character (blue line) at representative electrodes for (A)** high, **(B)** medium, and **(C)** low reading ability groups.

**Figure 2** shows the grand-averaged ERMs waveforms elicited by RC, PC, and NC in represented electrodes for children with different levels of reading ability. The typical N1, P200, and N400 patterns can be identified for every condition across the three reading-ability groups. The main interest of this study was the N400 from 300 to 600 ms. To obtain a fine-grained analysis of the time course of the N400 effect, the evaluation of N400 effect was subdivided into two time windows, including the time window of 300–450 ms (to capture the upslope and onset of the effect) and the time window of 450–600 ms (to capture the downslope and offset of the effect) for statistical analyses, as suggested by a previous study (Kreher et al., 2008).

We performed the cluster-based random permutation analysis (Maris and Oostenveld, 2007) to illustrate the topographic distributions of two types of lexicality effects in children with different levels of reading ability. Participants were sub-divided into three reading-ability groups based on their GCCRT scores (see **Table 1**). Mean amplitudes of two epochs (from 300 ms to 450 ms and from 450 ms to 600 ms) were analyzed separately in each reading ability group using the following procedures. The analytic steps were as follows. First, a simple dependent-samples *t*-test for each contrast (NC vs. RC, and NC vs. PC) was performed at each electrode. All electrodes that exceeded a significance level (α = 0.05) were identified and formed clusters. For each cluster, a cluster-level test statistic was calculated by taking the sum of all the individual *t*-statistics within that cluster. Then, a null distribution was created by computing 1000 randomized cluster-level test statistics. Finally, the actually observed cluster-level test statistics were compared against the null distribution and only clusters falling in the highest or lowest 2.5th percentile were considered significant. This procedure allows for the identification of the spatial distribution of lexicality effects and could effectively handle the multiple-comparisons problem.

#### ERPs Data: Linear Mixed Model Analysis

In the cluster-based random permutation analyses, there was a priori assumption that lexicality effects would be modulated by reading ability, so children were divided into groups before performing the analysis. Therefore, we further applied stepwise regression analyses with LMM method to ascertain whether the GCCRT scores and its interaction with the two types of lexicality effects have unique and significant relationships on N400. Specifically, LMM models were used with the backward elimination procedure. Each LMM model included participants and items as random effects. For fixed effects, the full model included continuous variables (digit span, phonological awareness, PPVT, GCCRT, age) and Lexicality (see Behavioral Data). Since age is highly correlated with GCCRT score, the continuous predictors (digit span, phonological awareness, PPVT, GCCRT, age) were centered by *z*-score transformation of raw score to overcome the problem of collinearity. Then, the less complex models, in which one fixed factor was excluded, would be compared to the full model. The log-likelihoods of the models were used to produce the chi-square statistics (Pinheiro and Bates, 2000). Predictors that did not lead to significant model improvement, based on chi-square statistics, were eliminated from the model. Meanwhile, it is unavoidable that multichannel EEG signals have significant correlation (Srinivasan et al., 2013). To include all spatial locations as a factor would introduce spatial autocorrelation in the residuals of statistical models of EEG data and thus violate the assumption of independent and identically distributed (i.i.d.) residuals (Dormann et al., 2007). Therefore, EEG electrodes were divided into six regions of interests (ROIs), including left-frontal (F7, FT7, T7, F3, FC3, and C3), central-frontal (FZ, FCZ, and CZ), right-frontal (F8, FT8, T8, F4, FC4, and C4), left-parietal (TP7, P7, CP3, and P3), central-parietal (CPZ and PZ), and right-parietal (TP8, P8, CP4, and P4). These ROIs were defined so that LMM analyses could be sensitive to lexicality effects in sub-regions of the scalp. The LMM analysis was applied separately with EEG data of each ROI and each time window.

The LMM analyses were conducted by using the lmer program of the lme4 package from in R 3.2.3 (R Core Team, 2015). In the final model, the significance of fixed effect was assessed by using the lmerTest package. In addition, we used the remef function (Hohenstein and Kliegl, 2013) to calculate adjusted means of ERP measures for each condition of each participant, by removing variance of random factors from the dependent variables. Finally, we conduct the correlation analyses to examine how GCCRT scores in relation to the adjusted ERP measures of each condition (NC, PC, and RC) and the two contrasts of interests (NC-PC and NC-RC).

## Results

### Behavioral Data

Trials with RT faster than 200 ms (3%) and slower than 3000 ms (2%) were excluded from models of RT. **Table 2** summarizes means and standard deviations of RT and the percentage of pronounceable responses in three groups, and **Table 3** presents the results of LMM analyses for RT and response preference. The LMM analysis on RT showed a significant High-Medium effect, indicating high-reading ability group revealed faster RT than medium-reading ability groups (*p* < 0.05). Significant effects of NC-RC and NC-PC contrasts on RT revealed that NC revealed faster RT than RC (*p* < 0.01) and PC (*p* < 0.001) did. However, no significant interactive effects were found in RT.

**Table 2:** The mean RT and percentage of pronounceability of ERPs experiment.

	The mean RT (ms)			The mean percentage of judging stimuli as pronounceable (%)		
	RC	PC	NC	RC	PC	NC
High ability	667 (207)	747 (201)	619 (136)	87 (12)	34 (21)	8 (15)
Medium ability	797 (241)	938 (291)	760 (199)	86 (12)	36 (23)	5 (5)
Low ability	871 (297)	925 (330)	767 (244)	73 (23)	33 (24)	9 (16)

*Standard deviations are given in parentheses. RC, real character; PC, pseudocharacter; NC, non-character.*

**Table 3:** Linear mixed model (LMM) estimates of fixed effects for RT and respond preference.

	Reaction times (ms)			Responses preference		
	Beta	Standard error	*t*-value	Beta	Standard error	*t*-value
(Intercept)	784.5	31.88	24.61	0.41	0.02	22.26
NC-RC	-59.1	17.81	-3.32**	-0.74	0.03	-27.3*
NC-PC	-152.20	17.81	-8.55***	-0.28	0.03	-10.19*
High-Medium	-154.26	74.67	-2.07*	0.01	0.05	0.172
Medium- Low	-11.81	80.72	-0.15	0.03	0.05	0.663
NC-RC × High-Medium	-9.58	41.70	-0.23	0.02	0.07	0.328
NC-PC × High-Medium	49.92	41.70	1.20	0.05	0.07	0.721
NC-RC × Medium-Low	58.09	45.08	1.29	-0.18	0.07	-2.597*
NC-PC × Medium-Low	-25.59	45.08	-0.57	-0.05	0.07	-0.746

*NC-RC, the contrast between non-character and real character. NC-PC, the contrast between non-character and pseudocharacter. High – Medium, the contrast between High ability and Medium ability. Medium – Low, the contrast between Medium ability and Low ability. NC-RC × High -Medium, the interaction between NC-RC contrast and High-Medium contrast. NC-PC × High -Medium, the interaction between NC-PC contrast and High-Medium contrast. NC-RC × Medium-Low, the interaction between NC-RC contrast and Medium–Low contrast. NC-PC × Medium–Low, the interaction between NC-PC contrast and Medium–Low contrast. ^∗^*p* < 0.05; ^∗∗^*p* < 0.01; ^∗∗∗^*p* < 0.001.*

For the response preference, significant effects of NC-RC and NC-PC contrasts (*p*s < 0.001) revealed that participants showed a higher preference for judging RC and PC as pronounceable than for NC. The significant interaction between Medium–Low contrast and NC-RC contrast suggested that, comparing to low-reading ability group, medium-reading ability group revealed a higher tendency to judge RC as pronounceable than for NC (*p*s < 0.05).

#### ERPs Data: Cluster-Based Random Permutation Analysis

**FIGURE 3:**
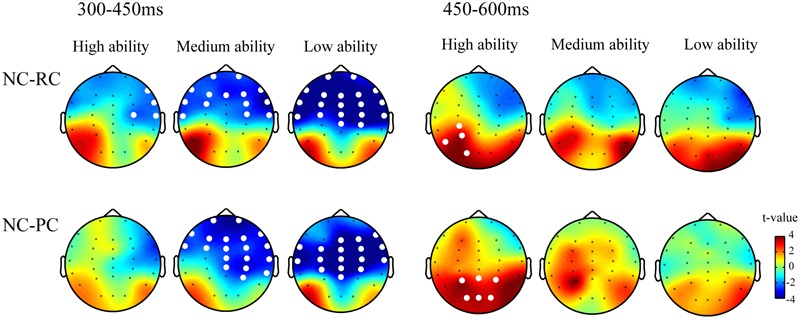
**Topographic maps for NC-RC and NC-PC contrasts in the early (300–450 ms) and late (450–600 ms) time windows.** White dots represent the electrodes that showed significant differences for the contrast.

**Figure 3** shows the results of the cluster-based random permutation analysis for both NC-RC and NC-PC contrasts in two time windows for high-, medium-, and low-reading ability groups. For the NC-RC contrast, both low- and medium-reading ability groups showed significant negative clusters in the frontal-to-central regions (*p*s < 0.01) in 300-450 ms and showed no significant cluster in the later time window. For the high-reading ability group, the NC-RC contrast also yielded a significant negative cluster that was restricted to the right frontal region between 300 and 450 ms (*p* < 0.05) and a significant positive cluster in the left parietal region (*p* < 0.05).

Similar findings were also observed in the NC-PC contrast. In the 300–450 ms, both low-and medium-reading ability groups (*p* < 0.0001 and *p* < 0.01, respectively) showed significantly negative clusters in the frontal-to-parietal regions. However, no significant cluster was found in high-reading ability group. In the later time window of 450–600 ms, high-reading ability group revealed a significant positive cluster in the central posterior region (*p* < 0.01), while no significant cluster was found in medium- and low-reading ability groups.

#### ERPs Data: LMM Analysis

##### 300–450 ms

For the LMM analysis of ERP data in 300–450 ms, the backward model selection revealed that digit span, PA, PPVT and age didn’t lead to significant model improvement. Therefore, only GCCRT, lexicality and the interaction between GCCRT and lexicality remained in the final model of each ROI. The results for each ROI are summarized in **Table 4**. The LMM analysis revealed significant NC-RC effects, in which NC elicited greater negativity of N400 than RC in left-frontal, central-frontal, right-frontal and central-parietal (*ps* < 0.01). On the other hand, left-parietal ROI revealed an opposite NC-RC effect (*p* < 0.0001) indicating that RC elicited greater negativity of N400 than NC did. Significant interactions between GCCRT and NC-RC contrast were found in left-, central-, and right-frontal ROIs (*ps* < 0.0001) and left- and central-parietal ROIs (*p*s < 0.01). The interaction effects suggested that the NC elicited greater N400 than RC did were more salient in children with lower GCCRT score. For the NC-PC contrast, it was significant in left-frontal, central-frontal, right-frontal, central- parietal and right-parietal ROIs (*p*s < 0.01), and that NC yielded more negative N400 than PC did. Moreover, interactions between GCCRT and NC-PC contrast were significant in all ROIs (*p*s < 0.01). The finding implied that the finding of NC elicited more negativity N400 than PC did was more salient in children with lower GCCRT score.

**Table 4:** Linear mixed model estimates of fixed effects for N400.

		300–450 m			450–600 ms		
ROI	Variables	Beta	Standard error	*t*-value	Beta	Standard error	*t*-value
Left -Frontal	(Intercept)	-1.65	0.25	-6.51	0.06	0.23	0.27
	NC-RC	-1.06	0.22	-4.92***	-0.02	0.20	-0.08
	NC-PC	-0.55	0.22	-2.55*	0.34	0.20	1.69
	GCCRT	0.09	0.24	0.37	0.31	0.22	1.41
	NC-RC × GCCRT	0.67	0.10	7.10***	0.28	0.09	3.21**
	NC-PC × GCCRT	0.51	0.10	5.42***	0.22	0.09	2.49*
Central -Frontal	(Intercept)	-2.55	0.35	-7.27	0.32	0.29	1.09
	NC-RC	-1.13	0.28	-4.59***	-0.46	0.26	-1.78
	NC-PC	-0.89	0.28	-3.16**	0.33	0.26	1.28
	GCCRT	0.47	0.34	1.40	–	–	–
	NC-RC × GCCRT	0.88	0.16	5.42***	–	–	–
	NC-PC × GCCRT	0.81	0.16	4.97***	–	–	–
Right -Frontal	(Intercept)	-1.55	0.30	-5.18	-0.10	0.22	-0.45
	NC-RC	-1.37	0.23	-6.02***	-0.71	0.19	-3.81***
	NC-PC	-1.12	0.23	-4.92***	-0.03	0.19	-0.16
	GCCRT	0.17	0.29	0.60	–	–	–
	NC-RC × GCCRT	0.48	0.09	5.08***	–	–	
	NC-PC × GCCRT	0.37	0.09	3.95***	–	–	–
Left -Parietal	(Intercept)	-0.35	0.25	-1.43	-0.48	0.23	-2.09
	NC-RC	0.57	0.17	3.33**	0.86	0.17	4.99***
	NC-PC	0.10	0.17	-0.59	0.80	0.17	4.65***
	GCCRT	-0.12	0.24	-0.49	0.14	0.22	0.64
	NC-RC × GCCRT	0.36	0.11	3.36***	0.12	0.10	1.18
	NC-PC × GCCRT	0.32	0.11	3.05**	0.28	0.10	2.86**
Central -Parietal	(Intercept)	-2.95	0.37	-8.01	–	–	–
	NC-RC	-0.59	0.30	-0.30	–	–	–
	NC-PC	-1.00	0.30	-3.18***	–	–	–
	GCCRT	0.41	0.36	1.15	–	–	–
	NC-RC × GCCRT	0.57	0.22	1.34	–	–	–
	NC-PC × GCCRT	0.57	0.22	2.53*	–	–	–
Right -Parietal	(Intercept)	-0.26	0.25	-1.06	-0.28	0.25	-1.14
	NC-RC	-0.19	0.18	-1.05	0.43	0.18	2.37*
	NC-PC	-0.47	0.18	-2.62**	0.85	0.18	4.74***
	GCCRT	-0.07	0.24	-0.29	0.08	0.24	0.34
	NC-RC × GCCRT	0.10	0.11	0.94	-0.11	0.10	-1.06
	NC-PC × GCCRT	0.35	0.11	3.22**	0.30	0.10	2.92**

*NC-RC, the contrast between non-character and real character. NC-PC, the contrast between non-character and pseudocharacter. GCCRT, the Grade Chinese Character Recognition test. NC-RC × GCCRT, the interaction between GCCRT and NC-RC contrast. NC-PC × GCCRT, the interaction between GCCRT and NC-PC contrast. ^∗^*p* < 0.05; ^∗∗^*p* < 0.01; ^∗∗∗^*p* < 0.001.*

**FIGURE 4:**
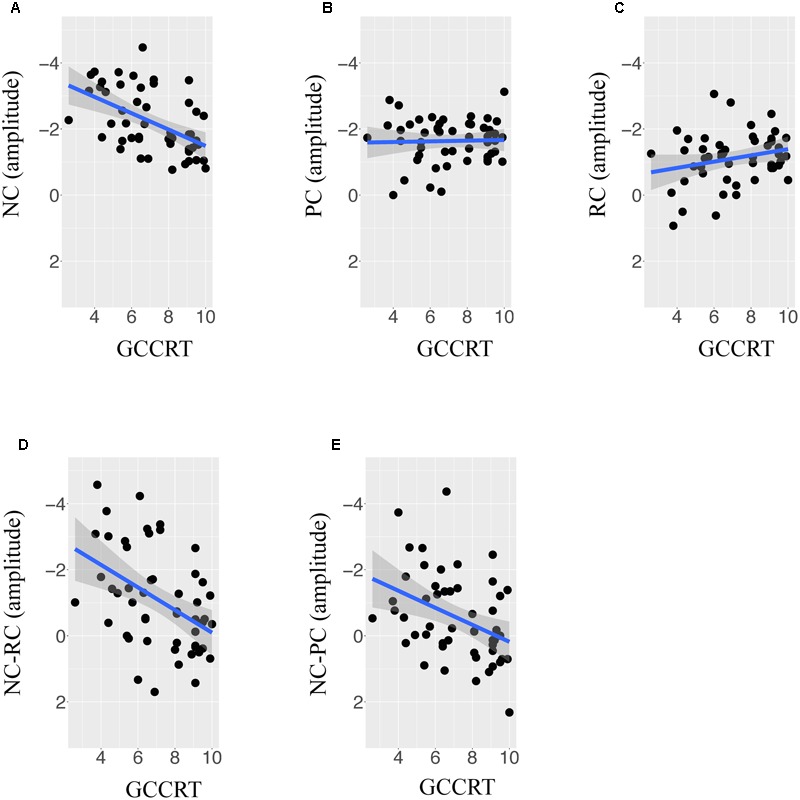
**The correlation between scores of Graded Chinese character recognition test (GCCRT) score and the adjusted mean amplitude of (A)** non-character, **(B)** pseudocharacter, **(C)** real character, **(D)** contrast of non-character versus pseudocharacter, and **(E)** contrast of non-character versus pseudocharacter at the left frontal ROI in the time window of 300–450 ms.

The observed relationship between lexicality effects and reading ability can be further supported by the correlational analysis. **Figures 4A–E** plots the mean amplitude of NC, PC, RC and two lexicality effects against standardized GCCRT score in the 300–450 ms time window. The trend lines showed the linear relationship between ERP measures and GCCRT scores, and the y axis is plotted negative-up. As shown in **Figures 4A–C**, GCCRT score was significantly correlated with the mean amplitude of NC (*r* = 0.52, *p* < 0.0001) but not with PC (*r* = -0.03, *p* > 0.1), and RC (*R* = -0.24, *p* > 0.05), respectively. These findings implied that children with higher GCCRT scores tended to show less negative amplitude to NC, and GCCRT did modulate N400 of RC and PC. **Figures 4D,E** further present correlational relationships between GCCRT and NC-RC contrast (*R* = 0.45, *p* < 0.0001) and between GCCRT and NC-PC contrast (*R* = 0.39, *p* < 0.01), respectively. Therefore, the interactions were due to more positive NC-PC and NC-RC effects for children with higher GCCRT score and more negative NC-PC and NC-RC effects for children with lower GCCRT score.

##### 450–600 ms

**FIGURE 5:**
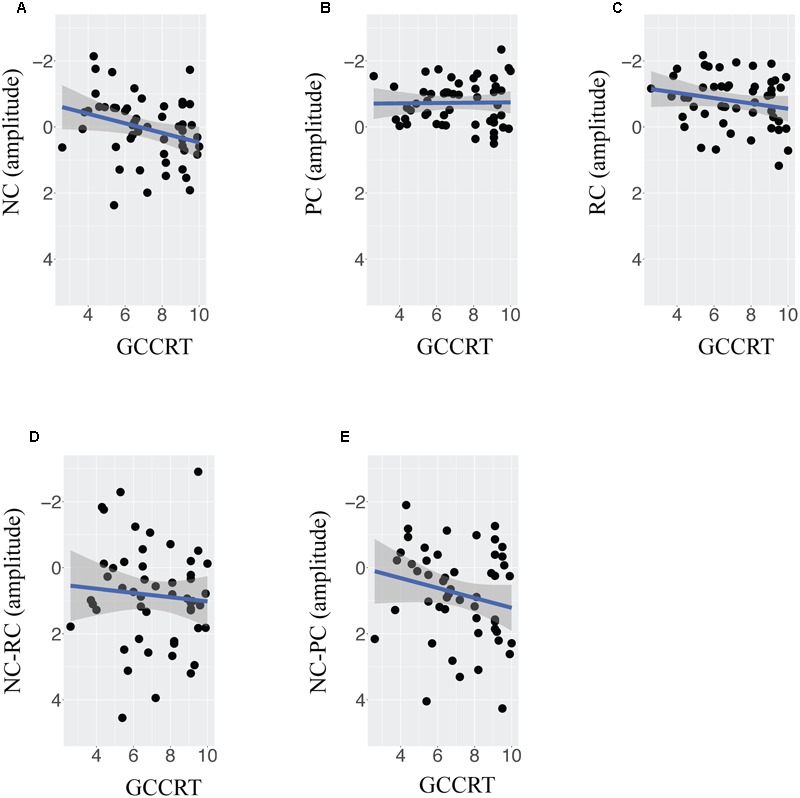
**The correlation between scores of Graded Chinese character recognition test (GCCRT) score and the adjusted mean amplitude of (A)** non-character, **(B)** pseudocharacter, **(C)** real character, **(D)** contrast of non-character versus pseudocharacter, and **(E)** contrast of non-character versus pseudocharacter at the left parietal ROI in the time window of 450–600 ms.

For the LMM analysis of ERP data in 450–600 ms of left-frontal, left-parietal, and right-parietal ROIs, the backward model selection revealed that digit span, PA, PPVT and age didn’t lead to significant model improvement. Therefore, only GCCRT, lexicality and the interaction between GCCRT and lexicality remained in final model of these ROIs. As for central- and right-frontal ROIs, only lexicality remained in the final model. Finally, none of the predictor remained in the final model in central-parietal ROI. The results for each ROI are summarized in **Table 4**. The NC-RC contrast was in right frontal ROIs (*p* < 0.001) and in left- and right-parietal ROIs (*p*s < 0.05). However, the interaction between GCCRT and NC-RC was only found in left frontal ROI (*p* < 0.01). The interactive effect suggests that NC tend to elicit more positive amplitude in this time window than RC did as children with better GCCRT scores. The significant NC-PC contrast, in which NC elicited more positive mean amplitude than PC, were found in left-and right parietal ROIs (*p*s < 0.001). Moreover, significant interactions between GCCRT and NC-PC contrast were found in left-frontal, left-parietal and right-parietal ROIs (*p*s < 0.05). These findings indicated that the greater positivity for NC than for PS in this time window increased with children’s GCCRT score. The correlational relationships between ERP measures in left-parietal ROI and GCCRT scores are presented in **Figures 5A–E**. The GCCRT score was significantly correlated with the mean amplitude of NC (*r* = 0.29, *p* < 0.05) in this time window. However, GCCRT did not show any significant correlation with PC (*r* = -0.02, *p* > 0.1), RC (*r* = 0.21, *p* > 0.1), NC-RC (*r* = 0.08, *p* > 0.1), and NC-PC (*r* = 0.21, *p* > 0.1).

## Discussion

This study aimed to use lexicality effects on N400 to investigate the development of orthographic knowledge in learning to read Chinese. Fifty-three typical developing children from the second to the sixth grades were asked to perform the pronounceability judgment task on a set of Chinese real characters, PCs and NCs, as ERPs were recorded. According to the literature, the typical finding for the lexicality effect in adults is that pseudowords elicit greater N400s than real words, while non-words do not elicit N400s but a greater positivity (Ziegler et al., 1997; Bentin et al., 1999). As more negative N400 has been associated with a greater effort in semantic retrieval or integration, absence of N400 elicited by non-words in adults suggests that they can decide that non-words violate the orthographic rules and are meaningless letter strings rather than potential words. Based on that, this study hypothesized that lexicality effects on N400 may reflect the acquisition of orthographic knowledge. That is, as children gain more reading experience with Chinese characters, they would gradually develop the concept of “word-likeness” and become more efficient in discriminating legal orthographic forms, such as characters and PCs, from the illegal ones, such as NCs. To be more specific, less experienced readers may treat all three types of stimuli as potential lexical items for lexical retrieval and show marked N400s to all types of stimuli. However, both NCs and PCs do not have the lexical entry in the mental lexicon and thus should elicit more negative N400 than real characters. As for more experienced readers, they should be able to identify NCs as illegal orthographic forms on the basis of visual and orthographic processing and thus revealed more adult-like lexicality effects on N400.

We first performed the cluster-based permutation analysis to characterize the spatial distributions of the two types of lexicality effects in children, as groups, with different levels of reading ability. The results seem to dissociate two sources of lexicality effects on N400. One at anterior sites in the early time window and the other one at central posterior sites in the late time window. Together with the results of correlational analysis, these findings support that reading ability shapes reader’s brain responses to NCs, which in turn modulates the two types of lexicality effects. Moreover, the reading ability modulates the two types of lexicality effect with different temporal dynamics and spatial distribution. Modulation effect on the greater negativity for NCs than for real characters was mainly found in the early time window with frontal distribution. As for the contrast between NCs and PCs, reading ability modulated such an effect in both time windows with reserved patterns and different topographic distributions. As we explained earlier, NCs also elicited greater negativity than PCs from centro-frontal to parietal sites. However, NCs became more positive than PCs did in the later time window and the modulation effect was mainly found in posterior region.

To further evaluate whether the pattern of lexicality effects would change with children’s reading ability, this study used the LMM analysis to take every participant’s age and their performance on a set of standardized behavioral assessments into consideration. The backward elimination procedure demonstrated that, GCCRT, rather than age, remain in the final LMM model. The finding is congruent with previous studies that orthographic sensitivity was mainly affected by reading achievement level, but not age (Allington, 1978; Coch et al., 2002). Meanwhile, the results of LMM analysis confirmed that NCs elicited a greater negativity than real characters did on N400, especially at the frontal ROIs. Its interaction with reading ability was mainly found in the early time window. These findings support that children with lower reading ability tend to treat NCs as potential lexical items and look for their meaning and thus show a greater effect than the children with higher reading ability. In addition, the LMM analysis also revealed a greater negativity for NCs than for PCs and its modulation effect with reading ability in 300–450 ms interval. However, the contrast between NCs and PCs revealed a reversed pattern in the later time window, in which the NCs elicited less negative mean amplitude than the PCs in left and right parietal ROI. Yet, the modulation effect with reading ability was only found at posterior sites. In other words, children with high reading ability tended to show a greater positive effect of differences between NCs and PCs at bilateral parietal sites. As shown in the grand ERP waveform, although high ability children also showed marked N400 to NCs in the early time window, the ERPs to NCs showed a positive tendency in the later time window, while the ERPs to PCs remain highly negative. That explains why the contrast of NCs and PCs showed reversed pattern in the early and late time windows, especially for children with high reading ability.

In addition to demonstrate the interaction between GCCRT scores and lexicality effects, we performed a correlation analysis between GCCRT scores and N400s of real characters, PCs, NCs and the two contrasts of interest in the representative regions that showed maximum modulation effect by the reading ability. In both time windows, GCCRT was positively correlated with the mean amplitude of NCs, but not with those of PCs and real characters. These findings support our main hypothesis. That is, children with lower reading ability have not yet mastered orthographic rules and thus treat NCs as potential lexical items to elicit greater N400. As they gain more reading experience, the negativity of N400 decreases with reading ability. Comparatively, N400s of RCs and PCs were not affected by reading ability. This explains positive correlations between reading ability and the two types of lexicality effects. That is, the greater negativity for NCs than that for real characters and PCs were more salient in children with low reading ability, especially in the early time window of N400.

It is worth noting that, both LMM and cluster-based analyses demonstrated that lexicality effects may be observed in frontal and posterior regions in early and late time windows of N400. These findings seem congruent with current suggestions for the dual mechanism underlying N400 for semantic processing (Lau et al., 2008; Wilson et al., 2012; Chang C.T. et al., 2016). Lau et al. (2008) proposed that N400 effects may originate from three brain regions as anatomical sources: the left posterior temporal cortex, the left anterior temporal cortex, and the left inferior frontal cortex. The posterior temporal cortex is responsible for storage of lexical-semantic information, and the anterior temporal cortex supports basic combinatorial operations for sentence processing. The inferior frontal cortex is involved in controlled retrieval and selection of lexical representations. This was supported by recent observations with aphasic patients, which showed that the topographic distribution of N400 effect can be modulated by patient’s language ability. For example, Wilson et al. (2012) observed a therapy-induced topographical change on N400 when aphasic patients were asked to determine whether spoken words were a “match” or “mismatch” to pictures of objects. Before therapy, nine aphasic participants exhibited a frontal-to-central maximum N400 mismatch effect over electrodes on the right hemisphere. After therapy, the N400 mismatch effect became left lateralized and maximized in central to posterior sites. The authors reasoned that the frontal-shifted N400 may reflect a change in the relative contribution of the neural generators underlying N400. Chang C.T. et al. (2016) examined the predictability effect on N400 in aphasic patients and found that the severity of comprehension deficit modulates the topographic distribution of the cloze probability effect on N400. To be more specific, aphasic patients with poorer reading comprehension, regardless of their diagnosis, tended to exhibit a reduced and delayed N400 effect of word predictability with a frontal scalp distribution. The anterior-shifted N400 in this study was interpreted as a greater reliance on frontal mechanisms, such as an elaborated and more effortful semantic retrieval mechanism, in patients with low reading comprehension ability.

Congruent with Coch’s series study, our data also showed a more frontal-distributed lexicality effect, especially for children with lower reading ability. By subdividing N400 into two time windows, the data also revealed a more adult-like lexicality effect with central-posterior distribution in the later time window, especially for children with higher reading ability. These findings are in line with the view of a dual mechanism underlying N400. To be more explicit, less experienced readers have not yet mastered the orthographic rules of Chinese characters at an automatic level. Therefore, a greater reliance on the frontal mechanism to effortful orthographic and semantic processing was observed. However, in the later time window, children with higher reading ability were able to process Chinese orthography at an automatic level and showed a greater positivity to NCs than to PCs.

Previous ERP studies have used different tasks to investigate children’s lexicality effect. For example, Ziegler et al. (1997) used the letter search task in which the response accuracy was not directly relevant to the manipulation of lexicality. Coch and colleague’s series studies mainly used the go/no-go semantic category task, in which participants were required to respond to animal names while the target stimuli (real word, pseudoword, non-word, and false-font) need no response. Therefore, none of these studies could discuss the lexicality effect on the behavioral data. In the present study, children were asked to perform the pronounceability judgment on the presented real characters, PCs, and NCs. This design is advantageous in investigating how reading ability shapes children’s response tendency and reaction times on the three types of stimuli.

The behavioral data showed that children revealed a higher preference to judge real characters and PCs as pronounceable than to NCs, regardless of reading ability. The only significant interaction effect was the “NCs versus real character × Medium-Low” interaction. The interaction might due to that, the difference of responses tendency for real character and NCs in low-reading ability group (73% vs. 9%) seem to be smaller than that in the medium-reading ability groups (86% vs. 5%). The reaction time data revealed that, across three types of stimuli, children became faster in making judgment as they became better readers. However, children are faster to respond to the NCs than to real characters and PCs, especially for children with lower reading ability. This seems to be in conflict with our conclusion from the ERPs data, which suggested that the low ability children tend to treat NCs as potential lexical items to look for their meaning and thus elicited a greatest N400. We reason the finding was due to the task demand of the pronounceability judgment. This task requires participants to decide whether they think the presented stimuli are pronounceable. Intuitively, the pronounceable and unpronounceable responses were considered as “Yes” and “No” responses, respectively. Therefore, to make a “No” response should take a longer RT than to make a “Yes” response did. However, given the majority of the stimuli were not characters, a more efficient way for children would be to look for whether the stimuli contain any clue for guessing the potential pronunciations. Since the NCs were created by combining an illegal radical and a radical in the illegal position, children will be easier to reject it, regardless if they treat it as a potential character or not. On the other hand, both characters and PCs contain a phonetic radical for children to look for the potential pronunciations and thus take a longer reaction time. To test this hypothesis, we calculated the mean RT of pronounceable and unpronounceable responses for PCs, as this is the only condition that provides a more comparable trial numbers for both types of decisions (the mean percentage of pronounceable response to PCs was 34%). The findings confirmed our speculation that, for PCs, reaction times for pronounceable decision for low- and medium-reading ability groups (988 and 943 ms) seem to be slower than that for unpronounceable decision (864 and 910 ms). Only the high-reading ability group showed a faster reaction time for pronounceable (655 ms) than that for unpronounceable (794 ms) judgments.

Although NCs showed faster reaction time than both real characters and PCs did, NCs elicited greater N400, especially for low reading ability group. This suggests that, although children might not know how to guess NCs’ pronunciation and thus tend to decide them as unpronounceable, the greater N400 reflected that they did not stop making sense of NCs. Similar discrepancy between reaction times and the amplitude of N400 have also been reported in previous studies. For example, words with larger orthographic neighborhood used to show faster speed in lexical decision and greater N400 than those with smaller orthographic neighborhood (Holcomb et al., 2002). Although concrete words are usually responded faster than abstract words, concrete words also tend to elicit a greater negativity of N400 than the abstract words do (Kounios and Holcomb, 1994; West and Holcomb, 2000). These findings were reasoned as that, larger amplitude N400 responses observed to words with larger orthographic neighborhood or high concreteness words suggest that these items generally evoke more activities in the semantic system, regardless of task demands.

## Conclusion

This study demonstrated developmental changes of the lexicality effects on N400. As children become more advanced readers, the N400 elicited by the NCs changes from being more negative in the frontal regions to becoming more positive in the posterior region than that elicited by real characters and PCs. These findings suggest that the time course and the topographic distribution of the lexicality effect on N400 can serve as ERPs markers to evaluate children’s development of orthographic knowledge. This may have important practical implications for identification of children with reading difficulties or for the intervention evaluation in the future.

## Author Contributions

Y-LT collected, analyzed and interpreted the data and wrote the paper. C-HH analyzed and interpreted the data and wrote the paper. Y-CH collected and analyzed data. C-YL interpreted the data and wrote the paper.

## Conflict of Interest Statement

The authors declare that the research was conducted in the absence of any commercial or financial relationships that could be construed as a potential conflict of interest.
